# Adjunctive optical and magnetic stimulation for venous and mixed etiology leg ulcers: protocol for the NAZARÉ multicenter randomized controlled trial

**DOI:** 10.1186/s13063-026-09859-1

**Published:** 2026-06-29

**Authors:** Sebastian Probst, Camille Saini, Alessio Stefanelli, André Frei, Ewa Klara Stuermer, Joachim Dissemond, Lars-Peter Kamolz, Benedikt Weber, Jean-Paul Lembelembe, Jürg Traber, Dieter Mayer, Thomas Zehnder, Dave Baer, Claudio Ruzza, Jürg Hafner

**Affiliations:** 1 Skin Ostomy and Wound Care, Geneva School of Health Science, HES-SO University of Applied Sciences and Arts, Avenue Champel 47, Geneva, Switzerland; 2 Care Directorate, Geneva University Hospitals, Geneva, Switzerland; 3 Faculty of Medicine, University of Geneva, Geneva, Switzerland; 4 Faculty of Medicine Nursing and Health Sciences, Monash University, Melbourne, Australia; 5 College of Medicine Nursing and Health Sciences, University of Galway, Galway, Ireland; 6 Geneva School of Health Science, HES-SO University of Applied Sciences and Arts, Geneva, Western Switzerland Switzerland; 7 Comprehensive Wound Center, Department of Vascular Medicine, Translational Wound Research, University Medical Center Hamburg-Eppendorf (UKE), Hamburg, Germany; 8 Department of Dermatology, Venerology and Allergology, University of Duisburg-Essen, Essen, Germany; 9 University Hospital Graz, Graz, Austria; 10 Department of Dermatology, Medical University of Vienna, Vienna, Austria; 11 Augustine Clinic, Malestroit, France; 12 Venenklinik Bellevue, Kreuzlingen, Switzerland; 13 Department of Dermatology, University Hospital Zurich, Zurich, Switzerland; 14 Spital Thun, Thun, Switzerland; 15 Medical Centre Cité Générations, Onex, Switzerland; 16 Outpatient Wound-Care, Jura Hospital, Delémont, Switzerland

**Keywords:** Venous leg ulcers, Adjunctive therapy, Biophysical stimulation

## Abstract

**Background:**

Venous leg ulcers (VLUs) and VLUs associated with peripheral arterial disease (PAD) are common, recurrent, and costly to manage. Adjunctive biophysical therapies may improve healing outcomes when combined with standard of care (SOC). Concurrent optical and magnetic stimulation (COMS) has shown promising mechanistic and preliminary clinical results but there is a lack of robust randomized controlled trials (RCTs).

**Method:**

This novel adjunct using concurrent optical and magnetic stimulation in a multicenter RCT in Europe (NAZARÉ) is a phase IV, post-market, multicenter, parallel-group, superiority randomized controlled trial conducted in Switzerland, France, Germany, and Austria. A total of 122 adults with VLU or mixed leg ulcers (ankle-brachial index > 0.5 and < 1.30 or ankle artery pressure > 60 mmHg), ulcer area 2–50 cm^2^, and ulcer duration > 30 days and < 2 years will be enrolled. Following a 2-week run-in to confirm < 30% area reduction under SOC, participants will be randomized 1:1 to SOC + COMS or SOC alone. The primary outcome is percentage wound area reduction (PWAR) at week 12, assessed by blinded evaluators using standardized digital planimetry. Secondary outcomes include complete closure, time-to-healing, pain, health-related quality of life, recurrence, and healthcare resource use. Missing outcome data will primarily be analyzed as observed and may be addressed using multiple imputation under the assumption of data being missing at random.

**Discussion:**

This trial will rigorously assess the effectiveness of COMS as an adjunct to standard care for venous and mixed leg ulcers. By targeting chronic wounds and using a pragmatic, multicenter design, it aims to generate robust, generalizable evidence. If effective, COMS could offer a non-invasive, accessible option to enhance healing outcomes and reduce the burden of chronic leg ulcers.

**Trial registration number:**

ClinicalTrials.gov NCT06528873. Registred on 26 July 2024, https://register.clinicaltrials.gov/prs/app/action/SelectProtocol?selectaction=Edit&sid=S000ERKB&uid=U0003LG4.

## Administrative information

Note: The numbers in curly brackets in this protocol refer to SPIRIT checklist.

**Table:** 

Title (1)	Adjunctive Optical and Magnetic Stimulation for Venous and Mixed Etiology Leg Ulcers: Protocol for the NAZARÉ Multicenter Randomized Controlled Trial
Trial registration	ClinicalTrials.gov: NCT06528873
Protocol version	Protocol version 1, version 13.10.2025
Funding	This study is funded by an unrestricted grant from Piomic Medical AG, Zurich, Switzerland.
Author details	Sebastian Probst, DClinPrac, MNS, RN, Full Professor of Tissue Viability and Wound Care Geneva School of Health Science, HES-SO University of Applied Sciences and Arts, Western Switzerland, Geneva Switzerland; Care Directorate, Geneva University Hospitals, Geneva, Switzerland; Faculty of Medicine, University of Geneva, Geneva, Switzerland; Faculty of Medicine Nursing and Health Sciences, Monash University, Australia; College of Medicine Nursing and Health Sciences, University of Galway, Ireland Email: sebastian.probst@hesge.ch ORCID Number: 0000-0001-9603-1570 Camille Saini, PhD Geneva School of Health Science, HES-SO University of Applied Sciences and Arts, Western Switzerland, Geneva Switzerland Email: camille.saini@hesge.ch ORCID Number: https://orcid.org/0000-0003-2459-3034 Alessio Stefanelli, MSc Geneva School of Health Science, HES-SO University of Applied Sciences and Arts Western Switzerland, Geneva Switzerland Email: alessio.stefanelli@hesge.ch ORCHID Number: https://orcid.org/0009-0009-0486-7414 André Frei, MSc Geneva School of Health Science, HES-SO University of Applied Sciences and Arts Western Switzerland, Geneva Switzerland Email: andre.frei@hesge.ch ORCHID Number: https://orcid.org/0009-0009-1378-4207 Ewa Klara Stuermer, MD, Professor University Medical Center Hamburg-Eppendorf (UKE), Department of Vascular Medicine, Comprehensive Wound Center, Translational Wound Research, Hamburg, Germany Email: e.stuermer@uke.de ORCID Number: 0000-0003-4193-4857, Joachim Dissemond, MD, Professor Department of Dermatology, Venerology and Allergology University of Duisburg-Essen, Essen, Germany E-Mail: joachim.dissemond@uk-essen.de ORCID-Nummer: 0000-0003-3893-328X. Lars-Peter Kamolz, MD, Professor University Hospital Graz E-Mail: lars.kamolz@medunigraz.at Benedikt Weber, MD PhD, Professor Department of Dermatology, Medical University of Vienna, Austria E-Mail: benedikt.weber@meduniwien.ac.at ORCID Number: https://orcid.org/0000-0002-7217-4590 Jean-Paul Lembelembe, MD Augustine Clinic E-Mail: jplembelembe@ghsa.fr ORCID Number: https://orcid.org/0000-0003-0405-2310 Jürg Traber, MD Venenklinik Bellevue Kreuzlingen, Switzerland E-Mail: j.traber@venenklinik.ch Dieter Mayer, MD, Privatdozent Department of Dermatology, University Hospital Zurich, Zurich, Switzerland E-Mail: dieter.mayer@usz.ch ORCID Number: 0000-0001-5933-7749 Thomas Zehnder, MD Spital Thun, Thun Switzerland Email: Thomas.Zehnder@spitalstsag.ch Dave Baer, MD Medical Centre Cité Générations, Onex, Switzerland E-Mail: Dave.baer@cpo.ch Claudio Ruzza, MD Outpatient wound-care, Jura Hospital, Delémont, Switzerland E-Mail: Claudio.Ruzza@h-ju.ch Jürg Hafner, MD, Professor, Vice Chair, Department of Dermatology, University Hospital or Zurich, Switzerland E-Mail: juerg.hafner@usz.ch ORCID Number: 0000-0002-4571-1143
Name and contact information for the trial sponsor	Sebastian Probst, Geneva School of Health Science, HES-SO University of Applied Sciences and Arts, Western Switzerland, Avenue Champel 47, CH-1206 Geneva, Switzerland, Tel: + 41 22 558 50 60, e-mail: sebastian.probst@hesge.ch
Role of sponsor	The study sponsor, Professor Sebastian Probst, will have ultimate authority over all aspects of the study, including its design, data collection, management, analysis, and interpretation. The sponsor will also oversee the preparation of the study report and will make the final decision regarding submission for publication. No external funders will influence any phase of the research process, analysis, or reporting.

## Introduction

### Background and rationale

Chronic leg ulcers (LU), particularly venous leg ulcers (VLUs), represent one of the most prevalent and persistent forms of lower extremity ulceration [1], with substantial impacts on patients’ physical health, quality of life, and economic well-being [2, 3]. VLUs account for up to 80% of all lower extremity ulcers and remain a leading cause of chronic wound care needs worldwide [4]. The global pooled point prevalence in the general population is estimated at 0.32% (95% CI, 0.11–0.60), and the annual incidence at 0.17% (95% CI, 0.08–0.29) [5]. Prevalence increases markedly with age, with older adults, particularly those aged ≥ 65 years, disproportionately affected. Recurrence is frequent, with up to 76% of patients experiencing ulcer recurrence within one year of healing, underscoring the chronic and relapsing nature of the condition [6].

The epidemiology of VLUs varies across regions, reflecting differences in population demographics, comorbidity profiles, and health system structures. High-income countries report prevalence rates between 0.1% and 0.6% in the general population, with rates exceeding 2% among individuals over 80 years of age [7]. Risk factors for recurrence include persistent venous reflux, inadequate compression maintenance, obesity, reduced mobility, and poor adherence to treatment regimens [3, 8]. Peripheral arterial disease (PAD) further complicates ulcer healing by reducing arterial inflow, leading to a mixed etiology ulcer with poorer prognoses and fewer effective treatment options [7].

The clinical and economic burden of VLUs is considerable, with estimated annual direct medical costs of approximately $10.73 billion (USD) across the studied geographies, averaging about $5,527 per patient per year for those managed conservatively [9]. In the UK, chronic wounds account for approximately 4% of total National Health Service spending, with VLUs representing one of the most resource-intensive categories [10]. Similar patterns are reported in the USA and Australia [11, 12]. Direct costs include dressings, compression systems, nursing visits, and hospital admissions, while indirect costs, such as work absenteeism, informal caregiving, and early retirement, add substantially to the societal impact [13].

Pathophysiologically, VLUs are driven by sustained venous hypertension, which leads among others to leukocyte activation, inflammatory mediator release, and microcirculatory dysfunction [1]. These processes impair the delivery of oxygen and nutrients essential for tissue repair [1]. In cases of VLU and PAD, impaired arterial perfusion exacerbates tissue hypoxia, further delaying healing [14]. Standard of care (SOC) for VLUs centers on compression therapy, wound bed preparation, infection control, and management of systemic comorbidities [12]. Compression therapy remains the gold standard of conservative treatment. However, its effectiveness is limited by inconsistent patient adherence, which is often hindered by discomfort, difficulty with application, and insufficient patient education [15–17].

Given these challenges, there is increasing interest in adjunctive therapies that target underlying pathophysiological mechanisms. Concurrent optical and magnetic stimulation (COMS) is a novel biophysical approach that integrates photobiomodulation in the red (630–680 nm) and near-infrared (800–860 nm) spectrum with low-intensity pulsed magnetic stimulation (1–100 mT). Photobiomodulation enhances mitochondrial oxidative metabolism via cytochrome c oxidase activation, increases adenosine triphosphate (ATP) production, and modulates inflammation through nitric oxide photodissociation. Low-intensity magnetic stimulation influences mechanosensitive ion channels, modulates vascular tone, and promotes angiogenesis [18–20].

Preliminary clinical data suggest that COMS may accelerate wound healing, reduce pain, and improve quality of life in patients with chronic wounds [21]. However, these findings are derived primarily from small-scale, uncontrolled studies with limited follow-up, leaving the magnitude and durability of any clinical benefit uncertain. There is a clear need for adequately powered, randomized controlled trials (RCTs) with rigorous methodology to assess the effectiveness of COMS as an adjunct to SOC.

The novel adjunct using concurrent optical and magnetic stimulation for venous leg ulcers in a multicenter randomized trial in Europe (NAZARÉ) has been designed to address this gap by evaluating whether the addition of COMS to SOC results in greater percentage wound area reduction (PWAR) at 12 weeks compared with SOC alone. Secondary objectives include assessing complete wound closure rates, time-to-healing, pain, health-related quality of life, recurrence rates, and healthcare resource utilization over a 24-week period. This trial is expected to provide robust, clinically relevant evidence that will inform clinical practice and healthcare policy in the management of VLUs and VLU + PAD.

### Objectives

The primary objective of the NAZARÉ trial is to determine whether SOC plus COMS achieves greater percentage wound area reduction (PWAR) at 12 weeks compared with SOC alone. Secondary objectives are to assess PWAR and complete wound closure at each visit, time-to-healing, pain, health-related quality of life, recurrence, and healthcare resource use over a 24-week period.

### Trial design

The NAZARÉ trial is a phase IV, post-market, multicenter, parallel-group, superiority, RCT with blinded outcome assessment conducted across European specialist wound care centers. It is designed to evaluate the efficacy of COMS when used as an adjunct to standard of care (SOC) in adults with VLUs or VLUs complicated by PAD.

## Methods: participants, interventions, and outcomes

### Study setting

The trial will be conducted at ten specialist wound care centers across Switzerland, France, Germany, and Austria. All participating sites have prior Good Clinical Practice (GCP)-compliant experience with interventional wound trials and will follow harmonized SOC protocols to minimize inter-site variability.

### Eligibility criteria

Inclusion criteria require that participants are aged 18 years or older, have a clinical diagnosis of VLU or VLU + PAD, and present with an ankle-brachial index (ABI) between 0.5 and 1.3 or an ankle artery pressure above 60 mmHg. Eligible ulcers must measure between 2 and 50 cm^2^ after debridement at screening and have been present for more than 30 days but less than 2 years. For patients with diabetes, the glycated hemoglobin (HbA1c) must be ≤ 12% at screening. All participants must provide written informed consent prior to any trial-specific procedure.

Exclusion criteria include pregnancy or lactation, active malignancy within the ulcer region, use of photosensitizing medication within the past 30 days, severe immunosuppression (including chronic corticosteroid therapy at > 10 mg/day prednisolone equivalent), New York Heart Association (NYHA) class III or IV heart failure, end-stage renal disease requiring dialysis, ulcer area reduction greater than 30% during the run-in phase, active infection requiring systemic antibiotics at baseline, use of advanced wound therapies (e.g., negative pressure wound therapy, skin substitutes, or hyperbaric oxygen therapy) within 2 weeks before screening, or participation in another interventional clinical trial within the past 30 days.

Eligibility criteria for study staff: Only investigators with experience in wound management and trained in COMS device use perform the intervention.

### Who will take informed consent?

Informed consent will be obtained by the site’s principal investigator or a study nurse who is GCP-certified. Potential participants will receive both verbal and written information about the study purpose, procedures, potential risks, benefits, and data confidentiality. Adequate time will be provided for questions before signing the consent form.

### Additional consent provisions for collection and use of participant data

If additional photographic data are collected for ancillary analyses or educational purposes, separate consent will be obtained specifying their scope, duration of storage, and anonymization procedures. Participants may opt out of future use without affecting trial participation.

### Explanation for choice of comparators

The comparator (SOC alone) reflects best practice for VLU management per international guidelines [22]. Its inclusion allows the effect of COMS to be isolated and compared against standard evidence-based wound care.

## Interventions

### Run-in phase

**Fig. 1:**
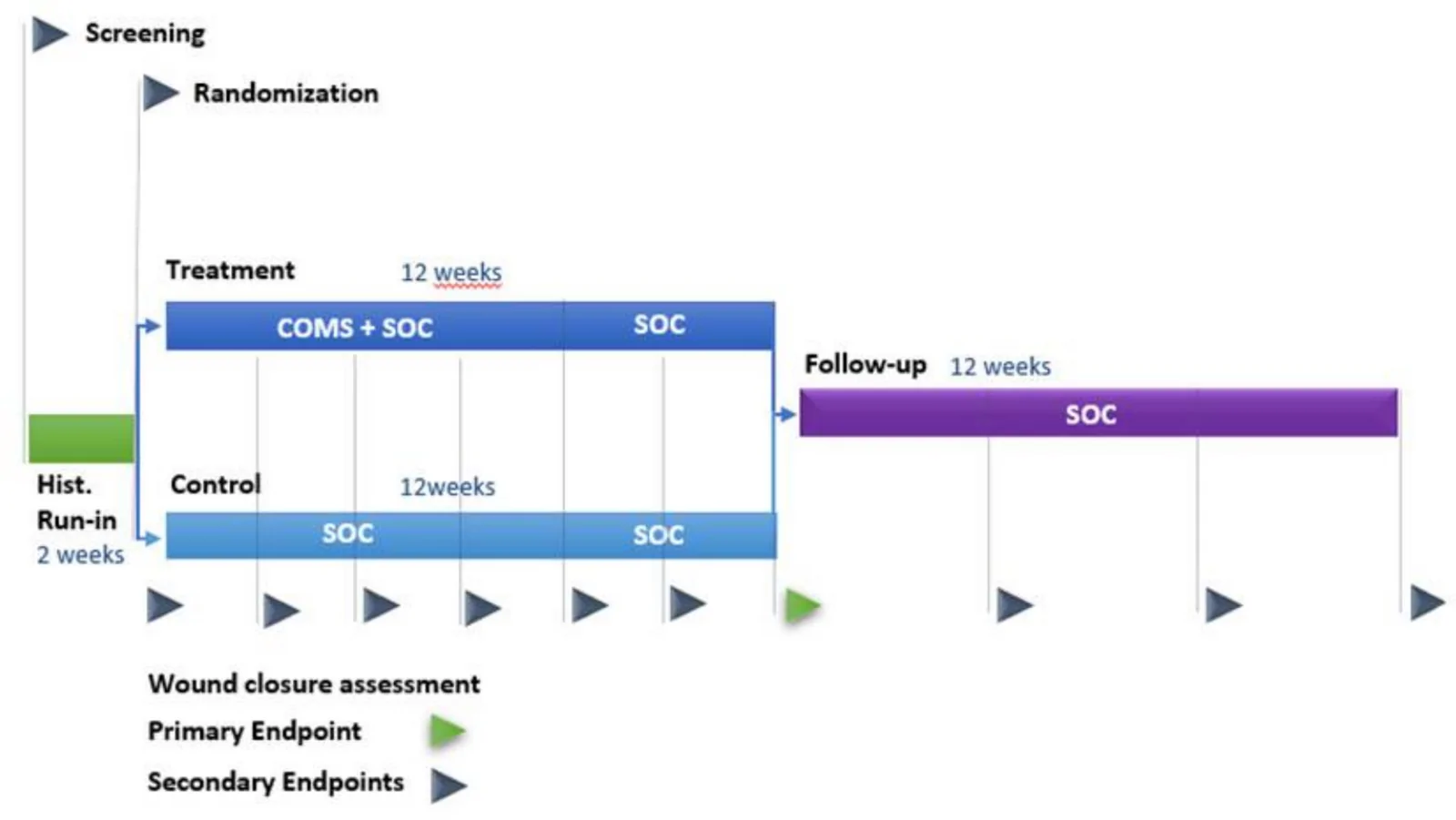
NAZARÉ study timeline with phases and endpoints schedule

A 2-week run-in period precedes randomization (see Fig. 1). During this phase, all participants receive optimized SOC, including wound cleansing, appropriate dressings, debridement when indicated, and compression therapy according to international guidelines. Wound area measurements are taken at screening and at the end of the run-in period. Participants whose ulcer area decreases by more than 30% during this period are excluded from randomization, as such wounds are likely to heal adequately with SOC alone. This strategy enriches the trial population with chronic ulcers and increases the study’s power to detect a true treatment effect.

### Standard of care (SOC)

SOC will be delivered in accordance with evidence-based guidelines [12, 23] and will be standardized across sites. This includes the use of multicomponent compression therapy to achieve an ankle pressure of 40–60 mmHg during the decongestion phase, followed by maintenance therapy with class II or III compression stockings. Wound care will focus on maintaining a moist wound environment, regular debridement, infection control, and optimization of systemic conditions such as glycemic control, blood pressure, and lipid profile.

### Experimental intervention (SOC + COMS)

The COMS device delivers combined photobiomodulation in the red (630–680 nm) and near-infrared (800–860 nm) wavelengths, together with low-intensity pulsed magnetic stimulation at field strengths of 1–100 mT [21]. Each treatment session lasts 16 min and is administered two to three times per week for 8 weeks [21]. The applicator is positioned 2–4 cm above the wound surface without direct contact. Calibration of light output and magnetic intensity is verified at production.

### Control arm (SOC only)

Participants receive optimized SOC identical to the experimental arm but without COMS treatment.

### Criteria for discontinuing or modifying interventions

Treatment discontinuation or modification will occur:

- Under emergency circumstances, based on his/her clinical judgment, the principal investigator at site may proceed to deviations from the CIP or study discontinuation to protect the rights, safety and well-being of the subjects, without prior approval by the Sponsor and the CEC. AEs related to the IMD and all SAEs must be reported and participants followed up until resolution of the event.
- If the scientific integrity of the investigation is compromised (noncompliance with the clinical trial protocol or GCP requirements from the part of the subject, the investigator, or the trial staff).
- If the participant decides to withdraw from the study.

### Strategies to improve adherence to interventions

When the intervention is under medical control, compliance is warranted. Device applications will be documented by clinical staff in the site device accountability log. When the intervention is performed in the homecare setting, participants will receive education on the rationale for session frequency, and the participant will be asked to complete a treatment diary. The planned treatment course consists of 24 sessions; completion of at least 16 sessions is considered the minimum exposure required to assess adherence, a minimum of 16 treatments. In case treatment sessions are missed, these can be made up in the 4-week follow-up.

### Relevant concomitant care permitted or prohibited

SOC compression and wound dressings are permitted; antimicrobial dressings may be used for local infection. Use of photosensitizing agents or medications and drug immunosuppression are prohibited. Arterial interventions, venous interventions, cell and tissue-based therapies (such as skin grafts), and advanced wound care therapies (such as negative pressure wound therapy) are prohibited. Concomitant medications and therapies for comorbidities, or any antibiotic treatments administered during the study period are allowed but must be documented accordingly.

### Provisions for post-trial care

Participants completing the study will return to usual clinical follow-up at their site. No post-trial access to COMS is planned, as the device is commercially available. Any adverse events related to the device and all SAEs that occur between the signing of the consent form and the end of the study will be followed up until resolution of the event. Compensation for harm will follow institutional policy and national law.

### Visit schedule

Participants attend visits at screening (week −2), baseline/randomization (week 0), and follow-ups at weeks 2, 4, 6, 8, 10, 12, 16, 20, and 24. Each visit includes ulcer assessment, wound photography, compression therapy documentation, changes in concomitant medications/therapies documentation, economic parameters documentation, and adverse event monitoring. Health-related quality of life and pain (Visual Analog Scale (VAS)) are assessed at baseline (week 0) and after 3 months (week 12). The 24-week visit assesses longer-term healing outcomes and ulcer recurrence.

### Ulcer measurement and photography

Ulcer area will be measured using standardized digital planimetry via the ImitoWound application [24]. Images are taken perpendicular to the wound, with a calibration ruler included in the frame. All wound images are uploaded to the central database for blinded review.

### Permitted and prohibited wound care products

A pre-approved formulary of dressings, including foam, hydrocolloid, alginate, and gelling fiber, is permitted. Antimicrobial dressings (e.g., silver) may be used only if there are clinical signs of local infection. Prohibited therapies during the trial include cell and tissue-based therapies (such as skin grafts, skin substitutes) and advanced wound care therapy (such as negative pressure wound therapy (NPWT)).

### Outcomes

#### Primary outcome

The primary outcome is PWAR at week 12 relative to baseline, measured via standardized planimetry with central blinded review.

#### Secondary outcomes

Secondary outcomes include PWAR and complete wound closure at weeks 4, 8, 12, 16, 20, and 24; time to 50%, 90%, and 100% wound area reduction; pain intensity (VAS) and health-related quality of life (Wound-QoL questionnaire) [25] at baseline (week 0) and after 3 months (week 12); ulcer recurrence within 24 weeks in participants whose ulcers achieve complete closure; healthcare resource utilization (dressings, debridement, consultations and COMS One applications); and device adherence and usability in the intervention arm.

#### Safety outcomes

All serious adverse events (SAEs) will be reported. Only adverse events (AEs) related to the investigational medical device (IMD) and not related to the natural course of VLUs evolution will be reported. Severity and connection to the IMD will be categorized according to ISO 14155 definitions.

### Participant timeline

Screening (week −2) → Baseline (week 0) → Follow-up visits at weeks 2, 4, 6, 8, 10, 12, 16, 20, and 24.

### Sample size

A total of 122 participants (61 per group) will provide 90% power to detect a 20% absolute difference in mean percentage wound area reduction (PWAR) at week 12 (65% in SOC + COMS versus 45% in SOC alone), assuming a standard deviation of 33%, a two-sided alpha of 0.05, and 5% attrition to account for potential loss to follow-up or drop-out [26, 27]. Sample-size calculations were performed using R (power.t.test). The assumptions were based on prior COMS pilot data and published SOC outcomes [21], where the mean wound area reduction with the COMS device was 70% (SD = 33%), and by two studies on SOC in chronic wounds [26, 27], where the mean reduction was approximately 45%. These data support the assumption of a 20% treatment effect in favor of COMS when added to standard care.

### Recruitment

Recruitment will occur consecutively at each site through outpatient wound care services and physician referrals. Sites will maintain a screening and enrollment log to track reasons for screening failures and randomization/intervention failures. Recruitment progress will be reviewed bi-weekly by the coordinating center.

### Assignment of interventions: allocation

Eligible participants will be randomized 1:1 to either SOC + COMS or SOC alone. The randomization sequence will be computer-generated by the study statistician using permuted blocks of variable sizes, stratified by site to maintain balance and minimize selection bias. Sequence generation will be performed using R with seed control and documented in a password-protected file accessible only to the statistician.

### Concealment mechanism

Allocation concealment will be ensured through the REDCap electronic data capture system, which automatically assigns treatment groups upon participant enrolment and eligibility criteria successful verification. The allocation sequence will remain concealed from site investigators until the system confirms randomization. No paper-based envelopes will be used.

### Implementation

The study statistician will generate the sequence; site investigators will enroll eligible participants; and REDCap will assign participants to groups automatically. This workflow maintains strict allocation concealment and traceability.

## Assignment of interventions: blinding

### Who will be blinded?

Due to the visible nature of COMS treatment, participant and clinician blinding is not feasible.

However, blinding will be maintained for outcome assessors, data analysts, and the steering committee. Wound images will be coded using participant’s unique identification numbers, and blinded assessors will review them centrally without knowledge of treatment arm or site. Statistical analyses will be conducted using masked treatment codes until database lock.

### Procedure for unblinding if needed

Unblinding will occur only if knowledge of allocation is essential for participant safety. The principal investigator may request unblinding via REDCap; the system will log all actions automatically. Outcome assessors will remain blinded whenever possible.

## Data collection and management

### Plans for assessment and collection of outcomes

Data will be recorded in REDCap, ensuring audit trails, role-based access, and secure encrypted storage. Ulcer size will be measured using standardized digital planimetry (ImitoWound), with calibration rulers and consistent lighting. Pain will be assessed via VAS; QoL via Wound-QoL questionnaire. Baseline variables (comorbidities, medications, ulcer history) will be collected through structured case report forms. Comorbidities collected include diabetes status and HbA1c, peripheral arterial disease, cardiovascular disease, hypertension, obesity (BMI), renal insufficiency, smoking status, and any condition associated with immunosuppression, as these are known to influence ulcer healing trajectories. Quality control checks will be implemented remotely after the inclusion of every 25 participants by an independent monitoring entity to identify and resolve discrepancies.

### Plans to promote participant retention and complete follow-up

Retention strategies include reminder calls for visits, flexible scheduling, and travel reimbursement (as permitted by local ethics). Participants who withdrew early will be invited for final data collection, and missing data will be minimized through prompt monitoring queries. Outcome data will be collected for all participants who discontinue intervention but consent to continued observation.

### Data management

Data entry will be performed by site staff and monitored remotely. Range checks and automated validation rules will be implemented in REDCap. All personal data will be pseudonymized using unique identifiers. The sponsor’s data management team will review cumulative data quality quarterly, and discrepancies will be resolved via Note-to-File/Deviation forms.

### Confidentiality

All participant data will comply with the GDPR and local data protection laws. Pseudonymized data will be stored on secure institutional servers; re-identification keys will remain locked separately at each site. Only authorized personnel will access identifiable data. Records will be retained for 20 years post-study completion in compliance with ISO 14155:2020. The NAZARÉ trial has received formal approval from the Geneva Ethics Commission (reference number 2024-D0096) and is registered at ClinicalTrials.gov (NCT06528873). All study procedures will comply with the Declaration of Helsinki (2013), ISO 14155:2020 for Good Clinical Practice in medical device investigations, and all applicable national and European regulations.

### Statistical methods

#### Statistical methods for primary and secondary outcomes

The primary endpoint (PWAR at week 12) will be analyzed using analysis of covariance (ANCOVA) adjusting for baseline ulcer area, ulcer type, and site. Binary outcomes (e.g., complete closure) will be assessed with logistic regression; time-to-event data (e.g., time to 100% closure) with Cox proportional hazards models; and repeated measures with linear mixed-effects models. The significance level is set at *α* = 0.05 (two-sided). Confidence intervals will be reported at 95%. All statistical analyses will be performed using Stata (version 16.1) and R (version 4.3.2).

### Methods for additional analyses

Exploratory subgroup analyses will assess treatment effects by ulcer etiology (VLU vs. VLU + PAD), baseline ulcer size, and center. Interaction terms will be included in the model; results will be interpreted cautiously as hypothesis-generating.

### Methods in analysis to handle protocol non-adherence and missing data

Analyses will follow the intention-to-treat principle. Missing outcome data will primarily be analyzed as observed and may be addressed using multiple imputation under the assumption of data being missing at random. Participants who deviate from protocol will remain in ITT unless withdrawn for safety.

### Interim analyses

No formal interim analysis or early stopping rules are planned due to the modest sample size and low-risk profile of the intervention. Descriptive safety summaries will be periodically reviewed by the sponsor.

## Plans to give access to full protocol, dataset, and statistical code

Upon study completion, anonymized participant-level data and statistical code will be shared in a public repository (Yareta) under FAIR principles, after publication of primary results. Access will require a data use agreement ensuring non-commercial reuse and participant confidentiality.

### Composition of the coordinating center and trial steering committee

The coordinating center (HES-SO Geneva) oversees day-to-day operations, data management, and site communication. The trial steering committee includes the sponsor (SP) and co-investigators (CS, AS, AF). They meet quarterly to review progress, protocol adherence, and safety. No separate endpoint adjudication committee is appointed; blinded outcome assessment substitutes for this function.

### Composition of data monitoring committee (DMC)

Given the minimal risk of COMS and its CE-marked status, a formal independent DMC is not required. Safety monitoring will be conducted by the sponsor and coordinating center according to ISO 14155 standards. Any unexpected device-related serious adverse events will trigger immediate review and reporting.

### Adverse event reporting and harms

All serious adverse events (SAEs) will be recorded and reported. Only adverse events (AEs) deemed device-related will be reported to authorities. Severity and causality will be graded per ISO 14155 definitions. Annual safety summaries will be submitted to ethics committees. Investigators will assess and document each (S)AE from onset until resolution.

### Frequency and plans for auditing trial conduct

Independent monitoring will be conducted after the enrolment of every 25 participants, reviewing consent forms, CRFs, and adverse event documentation. The audit will be independent of investigators and sponsor staff, and reports will be archived electronically.

### Plans for communicating important protocol amendments

Any substantial amendments (e.g., changes in eligibility criteria, outcomes, or statistical methods) will be communicated promptly to ethics committees, trial registries (ClinicalTrials.gov), and all investigators. Updated versions will include version number and date. Summary of modifications and ethics committees’ decision will be sent to all investigators for filing in the Investigator Site File (ISF) binder.

### Dissemination plan

Results will be disseminated through peer-reviewed journals and scientific conferences regardless of outcome. Plain-language summaries will be distributed to participants and patient associations. An anonymized dataset and statistical code will be publicly accessible following publication. All publications will acknowledge the funding source and follow ICMJE authorship criteria.

## Discussion

This study is designed to rigorously assess the efficacy of COMS as an adjunct to SOC in venous and mixed-etiology leg ulcers. These conditions represent a substantial and persistent challenge in wound management, with high prevalence, prolonged healing times, and frequent recurrences, all of which translate into a significant personal and societal burden [3]. Even with optimal SOC, including compression therapy, a substantial proportion of ulcers remain unhealed for extended periods, and recurrence is common within months of closure [28, 29].

One of the distinctive features of the NAZARÉ trial is the incorporation of a 2-week run-in phase prior to randomization. This methodological choice is based on evidence that early healing trajectories can predict eventual outcomes, and that wounds demonstrating rapid healing under SOC alone are less likely to benefit from additional interventions. By excluding ulcers that reduce in size by more than 30% during the run-in period, the study enriches its population for chronic ulcers, thereby improving statistical efficiency and increasing the likelihood of detecting a true treatment effect. This design also reduces heterogeneity in the study population, which is especially important in multicenter, international trials.

The pragmatic elements of the trial, such as allowing SOC to be delivered within harmonized but flexible guidelines and conducting the study in diverse clinical settings, enhance external validity. This approach ensures that the results, if positive, are generalizable to real-world practice across healthcare systems with varying resources and workflows. However, pragmatism also comes with trade-offs, including the inability to fully standardize all aspects of care and the potential for performance bias due to the absence of blinding among patients and treating clinicians.

If COMS demonstrates efficacy, it could be integrated into outpatient and community wound care settings as a non-invasive, well-tolerated adjunct to compression therapy. The portability and relatively short treatment duration make COMS compatible with home-based care, which could further improve accessibility and adherence. Particularly relevant in this context are VLU patients with an arterial component, where PAD limits the safe application of compression to the level that the venous component of the ulcer would otherwise require. For these patients, novel physical therapies may be especially important to compensate for the restricted use of high compression.

Future research could focus on identifying specific patient subgroups, such as those with mixed etiology LUs, recurrent disease, or poor compression adherence, who might derive the greatest benefit. In addition, cost-effectiveness analyses would be essential to inform healthcare policy and reimbursement decisions.

Conversely, if COMS does not demonstrate a clinically relevant benefit, the findings will still provide valuable guidance by redirecting resources towards more promising interventions, refining patient selection criteria, and improving trial designs for advanced wound care technologies. Regardless of the outcome, the NAZARÉ trial will contribute robust, high-quality evidence to an area of wound care where meaningful data are currently largely lacking.

## Trial status

Protocol version 2.1, dated 14 August 2025. Recruitment began in Switzerland in October 2025, with anticipated completion in December 2027.

## Data Availability

Upon study completion and publication of primary results, anonymized participant-level data and statistical code will be made available through the Yareta public data repository in accordance with FAIR principles. Data access will require a data use agreement ensuring non-commercial reuse and participant confidentiality.

## Abbreviations

ABI: Ankle–Brachial Index
AE: Adverse event
ANCOVA: Analysis of covariance
ATP: Adenosine triphosphate
CIP: Clinical Investigation Plan
CEC: Cantonal Ethics Commission
CE: Conformité Européenne
COMS: Concurrent Optical and Magnetic Stimulation
CRF: Case report form
DFU: Diabetic foot ulcer
DMC: Data Monitoring Committee
EC: Ethics Committee
FAIR: Findable, Accessible, Interoperable and Reusable
GCP: Good Clinical Practice
GDPR: General Data Protection Regulation
HbA1c: Glycated hemoglobin
HRQoL: Health-related quality of life
ICMJE: International Committee of Medical Journal Editors
IMD: Investigational medical device
IRB/REC: Institutional Review Board / Research Ethics Committee
ISF: Investigator Site File Investigator Site File
ISO: International Organization for Standardization
ITT: Intention-to-treat
LU: Leg ulcer
MAC: Medical adaptive compression system
MCS: Medical compression stockings
NPWT: Negative pressure wound therapy
NYHA: New York Heart Association (Heart Failure Classification)
PAD: Peripheral arterial disease
PCB: Phlebological compression bandages
PWAR: Percentage wound area reduction
QoL: Quality of life
RCT: Randomized controlled trial
SAE: Serious adverse event
SD: Standard deviation
SOC: Standard of care
SOP: Standard operating procedure
SPIRIT: Standard Protocol Items: Recommendations for Interventional Trials
USD: United States Dollar
VAS: Visual Analog Scale
VLU: Venous leg ulcer
Wound-QoL: Wound Quality of Life Questionnaire
